# Impact of virtual appointments on referral-to-appointment times for type 2 diabetes patients in Northwest London

**DOI:** 10.1038/s44401-026-00112-0

**Published:** 2026-07-01

**Authors:** Reham Aldakhil, Geva Greenfield, Gabriele Kerr, Alex Bottle, Benedict Hayhoe, Holger Kunz, Ana Luisa Neves

**Affiliations:** 1 Department of Primary Care and Public Health, Imperial College London, London, UK; 2 Institute of Health Informatics, University College London, London, UK

**Keywords:** Diseases, Health care, Medical research

## Abstract

Virtual healthcare delivery has become increasingly integrated into type 2 diabetes (T2D) management, but evidence on whether timeliness benefits are equitably distributed is limited. This study aimed to evaluate the impact of virtual versus in-person specialist appointments on timeliness of care for people with T2D and examine equity across demographic groups. We conducted a retrospective cohort analysis of outpatient appointments in Northwest London (January 2021–December 2024). The primary outcome was median referral-to-appointment (RTA) time. Wilcoxon rank-sum tests compared RTA times between virtual and in-person appointments, stratified by patient demographics. Benjamini-Hochberg correction controlled for multiple testing. Temporal trends were examined through year-by-year comparisons. Among 12,474 appointments (90.3% in-person, 9.7% virtual), virtual appointments were associated with 44% shorter median RTA time (20 vs 36 days, p < 0.001). Significant reductions were observed for both genders (males: 18.5 days shorter; females: 17 days), middle-aged and older adults (40-95 years: 15 days; 60-79 years: 22 days), Asian/Asian British patients (26 days), White patients (13 days), and across all deprivation quintiles, including the most deprived (IMD 1: 23 days, 59% reduction) and the least deprived (IMD 5: 26 days, 81% reduction). However, no significant differences were found for young adults (18-39 years), oldest adults (80+ years), Black/Black British, Mixed ethnicity, and other ethnic groups (all p > 0.05). Temporal analysis showed virtual RTA decreased from 35.5 to 8 days (2021-2024), while in-person RTA increased from 28 to 42 days. Virtual appointments were associated with improved timeliness of care for people with T2D overall when compared with in-person visits. However, this observational analysis does not establish causation, and further research (particularly prospective or experimental studies) is needed to confirm these relationships. Notably, important variations emerged across groups, underscoring the need for healthcare organisations to implement equity monitoring to ensure that any benefits are realised across all populations.

## Introduction

Type 2 diabetes (T2D) affects 4.9 million people in the UK and represents a major public health challenge^1,2^, with marked socioeconomic and ethnic disparities in both prevalence and outcomes^3,4^. While most T2D management occurs in primary care, patients with complex or poorly controlled diabetes require referral to specialist secondary care services for multidisciplinary assessment^5–7^. As T2D management becomes increasingly technology-enabled, understanding how different care delivery modes affect access to timely care across diverse patient populations are essential. Virtual care delivery has become an integral component of T2D management, offering the potential to improve timeliness through reduced travel burden, flexible scheduling, and efficient resource utilisation^8,9^.

Referral-to-appointment time (RTA), the interval from referral to first appointment date, serves as a key timeliness metric in the UK National Health Service (NHS), with an 18-week standard from primary care referral to a specialist appointment. Previous studies have examined telemedicine adoption, satisfaction, and clinical outcomes^9–11^, but few have rigorously quantified timeliness outcomes or systematically assessed whether efficiency gains are equitably distributed across diverse patient populations.

Current clinical practice increasingly relies on hybrid models combining virtual and in-person care, with virtual consultations now embedded across the series of patient care from initial assessments to ongoing medication adjustment and complication monitoring^12^. Professional societies, including the American Diabetes Association, now incorporate telehealth and virtual consultations as evidence-based care delivery options within their clinical guidelines^13,14^. The COVID-19 pandemic accelerated adoption of virtual consultations, understanding how timeliness benefits have evolved as services matured is therefore important.

However, this promising adaptation is counterbalanced by gaps in evidence. Studies have reported mixed experiences regarding timeliness, while some show reduced waiting times with virtual care, others document patient concerns about scheduling difficulties and unclear wait times^15,16^. In addition, concerns exist that virtual care may compromise equity through differential access, literacy, and quality of care across patient populations^17–20^. Importantly, pursuing efficiency gains through remote care delivery may inadvertently create a two-tier system: one where digitally literate, well-resourced patients benefit from convenient, timely virtual access while disadvantaged populations face longer delays, reduced access to hands-on examination, or forced adoption of modalities that do not meet their needs or preferences^19,21,22^.

This study evaluates the impact of virtual versus in-person appointments on timeliness for Type 2 diabetes patients, examining temporal patterns, and assessing equity by investigating whether timeliness varies by patient sociodemographic characteristics.

## Methods

### Study design

This retrospective cohort study analysed routine clinical data from specialist T2D services in Northwest London, UK, covering the period from 1 January 2021 to 31 December 2024. The study was conducted within an integrated care system serving a population of approximately 2.3 million residents across eight boroughs, characterised by substantial ethnic and socioeconomic diversity. This study adheres to the Strengthening the Reporting of Observational Studies in Epidemiology (STROBE) guidelines for reporting cohort studies. A completed STROBE checklist is provided as supplementary (supplementary 2)^23^.

### Data sources and variables

Data were sourced from the Whole Systems Integrated Care (WSIC) database, which links coded pseudonymised data for over 2.7 million patients registered with general practitioner (GP) practices in Northwest London. The dataset includes linked primary and secondary care records, including specialty outpatient appointments^24^. Variables extracted from the database are presented in the Supplementary Table S1. From 13,198 initial records, 724 (5.5%) were excluded due to data entry error (referral dates after appointment dates, *n* = 689, 5.2%), or extreme outliers (RTA > 365 days, *n* = 35, 0.3%).

### Population

The study included adult patients aged 18 years or older with a recorded diagnosis of T2D who were referred to specialist secondary care diabetes services between January 1, 2021, and December 31, 2024. Patients were identified using ICD-10 coding and referral pathway data extracted from electronic health records and gender was extracted from electronic health records as recorded at GP registration^25^. Appointments were included if they were an attended initial specialist appointment (excluding follow-ups) during the study period and had complete referral and appointment date information. Records where referral dates occurred after appointment dates were assumed to represent a data entry error and were therefore excluded.

### Primary outcome

The primary outcome was the referral-to-appointment (RTA) time, defined as the number of days between the referral request received date and the first appointment date, consistent with the NHS referral-to-treatment pathway measurement framework^26^. Outpatient appointment mode was classified as either in-person or virtual (i.e., telephone or video consultations)^27,28^.

### Statistical analysis

Patient characteristics were gender, age group, ethnicity, deprivation level (Index of Multiple Deprivation (IMD) Quintile)), and number of long-term conditions (LTCs). The number of LTCs was categorised as 1, 2, or 3+ conditions. Clinical complexity was quantified using the Charlson comorbidity index with England-validated contemporary weights^27,28^.

In line with the right-skewed distribution of RTA data, medians and interquartile ranges (IQR) as primary measures were reported. The 25th percentile (Q25), median (Q50), and 75th percentile (Q75) were calculated for each appointment mode within each demographic group. For each patient group, we calculated the difference in median RTA between in-person and virtual appointments, (1) Δ Median RTA = Median RTA (in-person) – Median RTA (virtual), with positive values indicating virtual appointments had shorter RTA, and (2) relative percentage differences in RTA were calculated.

For each demographic stratum, Wilcoxon rank-sum tests compared RTA times between virtual and in-person appointments^29,30^. Given the 20 demographic comparisons performed, Benjamini-Hochberg false discovery rate (FDR) correction was applied to control for multiple testing, both adjusted *p*-values and unadjusted values are reported, with a two-tailed significance threshold of q < 0.05 (FDR-adjusted)^31^.

Temporal analysis examined year-by-year trends from 2021 through 2024, comparing median RTA between in-person and virtual appointments for each calendar year. The study period was classified into phases: 2021 (pandemic with service disruption), 2022 (transition/recovery), 2023 (post-pandemic), and 2024.

To formally test whether the association between appointment mode and RTA differed by comorbidity burden, we fitted a linear regression model with RTA as outcome. Linear regression was used for CCI specifically because it permits formal testing of an interaction between a continuous comorbidity measure and appointment mode; this is not feasible with the stratified Wilcoxon rank-sum tests used for the other, categorical demographic characteristics. Regression coefficients, 95% confidence intervals, and *p*-values are reported in Supplementary Table S3.

A small proportion of records (5%) with missing information on age, gender, ethnicity, or LTCs were excluded from the stratified analyses. Complete data were available for all included appointments regarding referral dates, appointment dates, and appointment mode. All analyses were conducted using R statistical software version 4.3.1^32^.

### Sensitivity analysis

A set of bootstrap confidence intervals (CI) with patient-level resampling to account for clustering of multiple appointments within patients (95% CI for all RTA estimates) was used. Patient-level adjusted analysis using cluster-adjusted CIs was performed, with sensitivity analysis restricted to one appointment per patient (n = 3209, first appointment chronologically). Detailed results of all sensitivity analyses are presented in the Supplementary Table S3.

#### Ethics

Approvals and permissions to access the Whole Systems Integrated Care (WSIC) datasets for the purpose of this study were granted by the Northwest London Sub-Data Research Access Group on 14th June 2024. This study used fully anonymised, routinely collected healthcare data and was conducted under the UK Health Research Authority guidelines, which do not require separate research ethics committee approval.

## Results

### Study population and appointment characteristics

A total of 12,474 appointments were included in the analysis. The cohort was predominantly male (60.1%, n = 7497), and nearly half of the subjects were aged 60–79 years (47.3%, n = 5900). The cohort was ethnically diverse, comprising Asian/Asian British (39%, n = 4859), White (32%, n = 4008), and Black/Black British (15%, n = 1900) individuals. Only 17% (n = 2123) resided in the most deprived areas (IMD 1), while 5% were in the least deprived (IMD 5). The study population had moderate-to-high comorbidity (mean Charlson score 3.3, SD 2.8, median 3.0, IQR 1.0-5.0, range 0-19), with about two-thirds of the cohort (67%, n = 8,318) had three or more long-term conditions.

Out of 12,474 first appointments across the four years period, most were delivered within Endocrinology services (72%, n = 8999), followed by General Medicine (7%, n = 839) and Diabetic Medicine (5%, n = 667). Non-consultant professionals delivered 7% of appointments (n = 898) and had the highest proportion of virtual consultations (25.7%) (Table 1).

**Table 1:** Study population characteristic**s**

Demographics	Total (n = 12,474)	In-person (n = 11,266)	Virtual (n = 1208)	P-value
**Age group, n (%)**				< 0.001
18-39 years	1026 (8.2)	923 (8.2)	103 (8.5)	
40-59 years	4242 (34.0)	3757 (33.4)	485 (40.1)	
60-79 years	5900 (47.3)	5413 (48.1)	487 (40.3)	
80+ years	1306 (10.5)	1173 (10.4)	133 (11.0)	
**Gender, n (%)**				0.083
Male	7497 (60.1)	6766 (60.1)	731 (60.5)	
Female	4977 (39.9)	4500 (39.9)	477 (39.5)	
**Ethnicity, n (%)**				0.571
Asian/Asian British	4859 (38.9)	4373 (38.8)	486 (40.2)	
White	4,008 (32.1)	3,643 (32.3)	365 (30.2)	
Black/Black British	1,900 (15.2)	1,712 (15.2)	188 (15.6)	
Mixed	450 (3.6)	408 (3.6)	42 (3.5)	
Other	864 (6.9)	785 (7.0)	79 (6.5)	
Unknown	393 (3.2)	345 (3.1)	48 (4.0)	
**IMD quintile, n (%)**				0.018
1 (most deprived)	2123 (17.0)	1943 (17.3)	180 (14.9)	
2	1622 (13.0)	1455 (12.9)	167 (13.8)	
3	3176 (25.5)	2862 (25.4)	314 (26.0)	
4	4947 (39.7)	4435 (39.4)	512 (42.4)	
5 (least deprived)	601 (4.8)	566 (5.0)	35 (2.9)	
**Number of LTCs, n (%)**				0.066
1	1741 (14.0)	1556 (13.8)	185 (15.3)	
2	2420 (19.4)	2155 (19.1)	265 (21.9)	
3+	8313 (66.6)	7555 (67.1)	758 (62.7)	

*LTCs* long-term conditions

Demographic and clinical characteristics of the cohort (*n* = 12,474 appointments), stratified by appointment mode. Includes age group, gender, ethnicity, deprivation quintile, and number of long-term conditions.

The majority of the appointments were in person (90.3%, *n* = 11,266), with virtual appointments comprising 9.7% of the total (*n* = 1208). Gender, ethnicity, and number of LTCs did not differ between appointment modes (all *p* > 0.05) (Table 1).

### Overall impact of appointment mode on RTA time

**Fig. 1:**
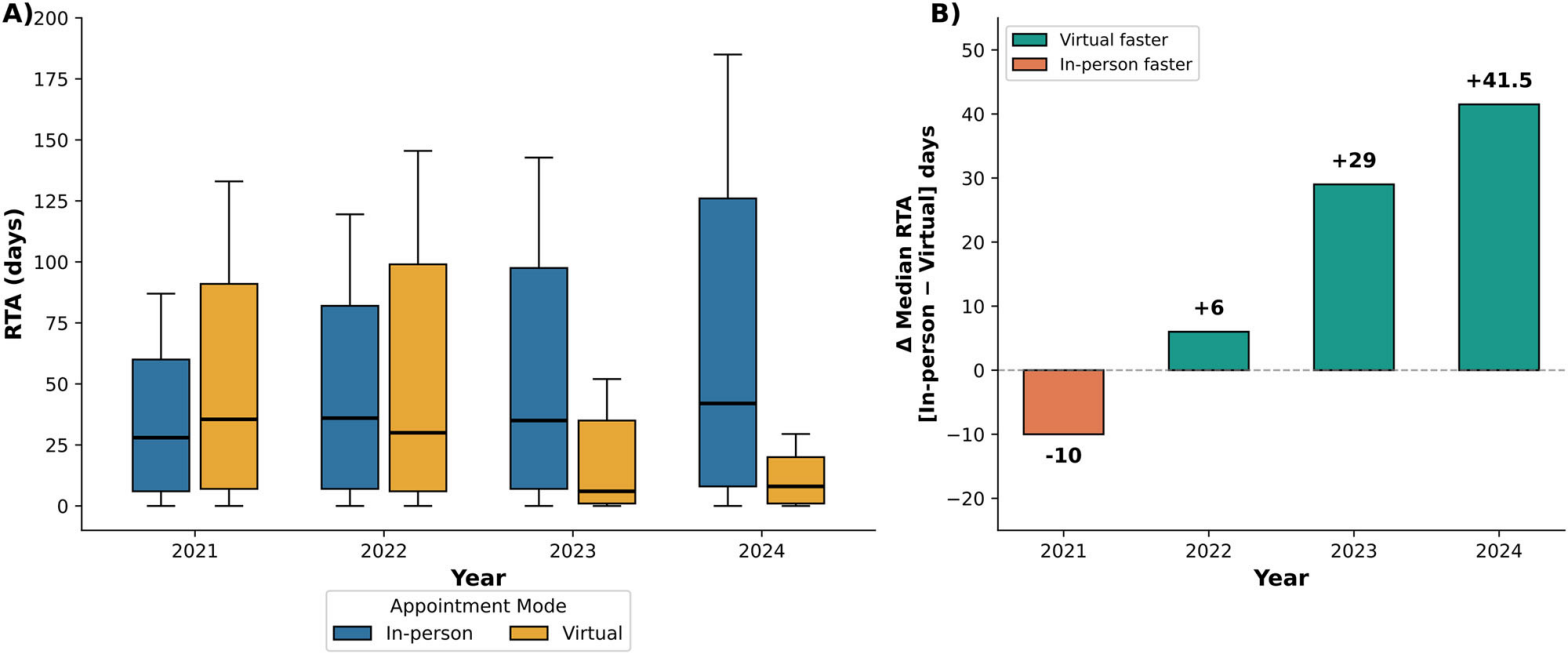
Year-by-year trends in referral-to-appointment time (RTA) by appointment mode (2021–2024). **A** Median RTA with interquartile range (IQR) for in-person (blue) and virtual (orange) appointments across four years. **B** Δ Median RTA (Median in-person RTA − Median virtual RTA) in days. Positive values (green bars) indicate virtual appointments had shorter RTA; negative values (red bars) indicate in-person appointments had shorter RTA. Data represent pooled analysis across the study period (*n* = 12,474 appointments).

Overall, virtual appointments were associated with significantly shorter median RTA time than in-person appointments (-16 days or 44.4% reduction, 20 vs. 36 days, p < 0.001) (Fig. 1). Virtual appointments also showed a lower variability in RTA times, when compared with in-person ones (IQR 76 vs. 91 days). After accounting for patients with multiple appointments, the difference remained robust: virtual appointments were 18 days shorter on average (95% CI: 13-23 days). Sensitivity analyses confirmed these findings. When restricted to one appointment per patient (n = 3209), the median RTA difference remained significant (22.5 days, 95% CI: 19–25 days).

### Changes in RTA times over time (2021–2024)

The relationship between appointment mode and RTA changed substantially over the study period (Fig. 1). Virtual appointments initially had longer median RTA than in-person appointments in 2021 (38 vs 28 days), but this pattern reversed in 2022 and continued through 2024.

By 2024, virtual appointments had a median RTA of 8.5 days compared with 50 days for in-person, a 41.5-day difference. These changes reflected simultaneous improvement in virtual RTA (77.6% reduction from 2021, IQR: 106 to 48 days) and deterioration in in-person RTA (78.6% increase, IQR: 55 to 141), with virtual appointments showing consistently lower variability. The median values fall towards the lower end of each IQR (Fig. 1), reflecting the right-skewed distribution of waiting times where most patients are seen relatively quickly but a minority experience substantially longer waits.

### Differential impact across patient groups

**Fig. 2:**
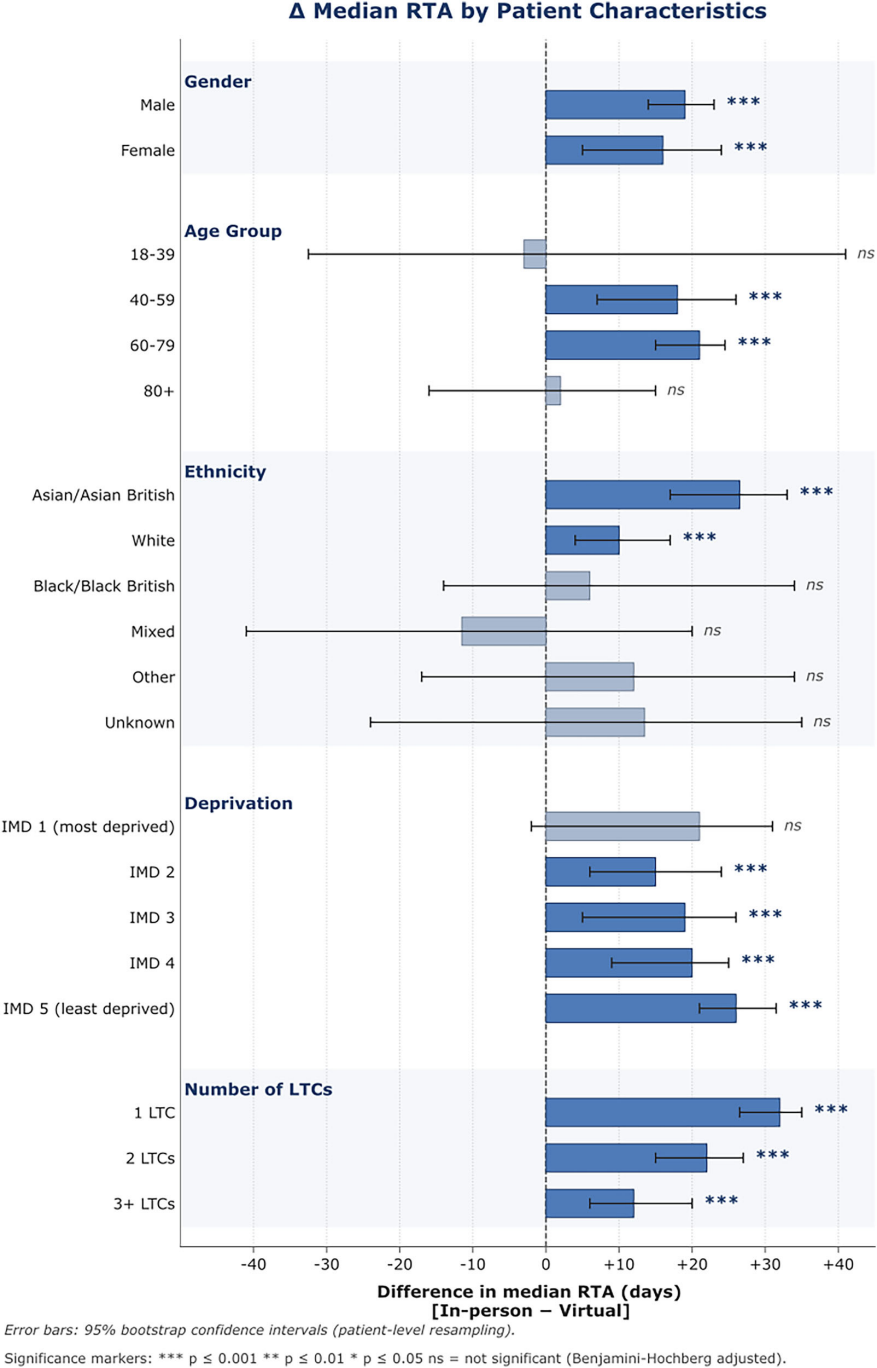
Δ Median RTA by patient characteristics. Difference in median RTA between in-person and virtual appointments (in days) stratified by gender, age group, ethnicity, deprivation quintile (IMD), and number of long-term conditions (LTCs). Positive values indicate shorter RTA with virtual appointments, negative values indicate shorter RTA with in-person appointments. Statistical significance assessed using Wilcoxon rank-sum tests with Benjamini-Hochberg correction (see Supplementary Table S2).

Overall, virtual appointments were significantly associated with shorter RTA times. However, important variation was observed for specific patient groups (Fig. 2, Supplementary Table S2). Patient-level analysis showed that both males (19 days,95% CI: 14-23 days) and females (16 days, 95% CI: 5-24 days) experienced significant RTA reductions when compared to in-person.

Adults aged 40-59 years (18 days, 95% CI: 7-26 days) and 60-79 years (21 days, 95% CI: 15-24.5 days) showed significant reductions in RTA times with virtual consultations, when compared to in-person care. However, no significant differences between modalities were observed for young adults aged 18-39 (median difference: -3 days, 95% CI: -33 to 41 days, p = 0.82) or older adults aged 80+ (2 days, 95% CI: -16 to 15 days).

Virtual appointments had significantly shorter RTA than in-person appointments for Asian/Asian British (26.5 days, 95% CI: 17-33 days, p < 0.001) and White patients (10 days, 95% CI: 4-17 days, p < 0.001). In contrast, no statistically significant differences in RTA times between virtual and in-person appointments were found for Black/Black British patients (6 days, 95% CI: -14 to 34 days), mixed ethnicity patients (-11.5 days, 95% CI: -41 to 20 days), other ethnic groups (12 days, 95% CI: -17 to 34 days), or patients with unknown ethnicity (13.5 days, 95% CI: -2524 to 35 days). Significant RTA reductions with virtual appointments were observed across most deprivation quintiles: IMD 5/least deprived (26 days, 95% CI: 21-31.5 days, p < 0.001), IMD 4 (20 days, 95% CI: 9-25 days, p < 0.001), IMD 3 (19 days, 95% CI: 5-26 days, p < 0.001), and IMD 2 (15 days, 95% CI: 6-24 days, p < 0.001). No significant differences between virtual and in-person were observed for those living in most deprived areas (IMD 1) (21 days, 95% CI: -2 to 31 days, p = 0.07).

Sensitivity analyses confirmed our findings, after Benjamini-Hochberg FDR correction for the 20 demographic comparisons, 13 of the 20 subgroups remained statistically significant (BH-adjusted p < 0.05), including both genders, adults aged 40–79 years, Asian/Asian British and White ethnicities, and IMD quintiles 2-5 (Supplementary Table S2).

The difference in RTA between virtual and in-person appointments was consistent across comorbidity levels; while the regression showed that higher Charlson scores were associated with significantly shorter RTA (p = 0.003), the interaction term was not statistically significant (p = 0.719) (Supplementary Table S3).

## Discussion

Virtual appointments were associated with waiting times nearly half as long as in-person (a 44% reduction); patients waited a median of 20 days compared to 36 days for in-person appointments, a difference that remained consistent across multiple statistical checks (18 days, 95% CI: 13-23 days). However, the observed improvements were inequitably distributed across patients’ groups. Significant reductions occurred for both genders, middle-aged and older adults (40-79 years), Asian and White patients, and moderately to least deprived areas. In contrast, seven groups showed no difference in RTA times between virtual and in-person appointments: young adults^18–39^, oldest adults (80 + ), Black/Black British, Mixed, and other ethnicities, and those in the most deprived areas (IMD 1). The gap between appointment types also widened dramatically over time: in 2021, virtual appointments had longer waiting times than in-person (35.5 vs 28 days), but by 2024, virtual waits had dropped to just 8 days while in-person waits had grown to 42 days.

Our finding that virtual appointments were associated with reduced median RTA by 16 days (44%) aligns with a recent systematic review by Capodici et al. (2025)^33^, reporting a weighted mean reduction of 25.4 days in waiting times with telemedicine implementation in Primary Care, with secondary care specialties showing reductions of up to 34.7 days^33^.

Our finding that virtual appointments had longer RTA than in-person visits in 2021, but progressively shorter RTA through 2022-2024, aligns with broader evidence of telemedicine maturation during this period. Cross-sectional studies consistently demonstrate that telemedicine reduces waiting times compared to in-person care^33,34^, while longitudinal research has documented steep learning curves for implementation of virtual consultations that are progressively overcome with time^9–11,35^. The OECD’s multinational survey documented substantial evolution in telemedicine infrastructure and practice between 2020-2022^34^. The temporal pattern observed in this study might reflect healthcare system learning, refinement of patient selection criteria, and optimisation of virtual care pathways.

The variation in benefit across ethnic groups warrant careful interpretation. Importantly, no ethnic group experienced significantly worse RTA times with virtual appointments compared to in-person care. Some ethnic groups have actually experienced significant benefits when compared to in-person care; this pattern is consistent with US telemedicine research. Bustamante et al. (2023) found that Hispanic and Black patients in California had significantly lower rate of telemedicine use before and after COVID-19 stay-at-home order than White patients, with language barriers and limited broadband access identified as key drivers of these differences. Other US studies have similarly documented lower telemedicine uptake among Black, Hispanic, and non-English-speaking populations, partly reflecting differential digital literacy and access to technology. In our cohort, the absence of a significant RTA benefit for Black/Black British patients may therefore reflect comparable structural barriers to telemedicine engagement in Northwest London, rather than a direct effect of appointment modality itself. This might be explained by variation in telemedicine use, as backed up by findings from US telemedicine studies documenting racial/ethnic variations in telemedicine use during the COVID-19 pandemic^36^.

Our finding that young adults aged 18-39 years showed no significant difference in RTA times between virtual and in-person appointments, is not in line with previous evidence suggesting that younger adults would have shorter RTA with telephone consultations^37,38^. The reasons for this are unclear, but may relate to differences in clinical presentation, referral urgency, or scheduling preferences^39,40^.

The socioeconomic gradient observed, where patients in the least deprived areas (IMD 5) showed a 26-days reduction, compared to no significant benefit for those in the most deprived areas (IMD 1), supports concerns that virtual care may exacerbate the inverse care law^41^. This finding is consistent with broader evidence that virtual care may compromise equity through differential access and quality of care across populations, and that the equity challenge in virtual care is driven primarily by access, digital literacy, language, and broadband, rather than by differential clinical effectiveness once access is achieved^16,19,39^.

Our findings revealed substantial variation in timeliness benefits across demographic groups, these disparities raise important equity concerns for healthcare systems expanding virtual care delivery.

From a policy perspective, these finding suggest that universal virtual-by-default approaches may exacerbate health inequalities. The 2019 NHS Long Term Plan committed to expanding remote care options^42,43^, but our results indicate that such expansion requires careful equity monitoring. Ethnic minorities (particularly Black and Mixed ethnicity patients) and socioeconomically deprived groups may require additional support to benefit equally from virtual care pathways.

Some patients with multiple conditions may benefit from virtual appointments due to difficulty attending in-person, while others may require in-person assessment regardless of comorbidity level^44,45^. Of note, comorbidity scores do not capture all factors relevant to appointment modality decisions; triaging patients’ suitability for virtual versus in-person consultations depends on clinical requirements that vary by specialty and individual presentation. The large waiting time difference between appointment types, combined with the low virtual care uptake (9.7% of appointments), suggests clinicians make complex allocation decisions based on multiple considerations our data cannot capture^46–48^.

From a practice perspective, GPs referring patients to specialist secondary care are well-positioned to indicate patient suitability for different appointment modalities. Including information in referral letters about communication difficulties, language needs, or patient preferences could help secondary care teams allocate appointments more appropriately.

Healthcare organisations should therefore establish real-time equity monitoring systems that track access and outcomes across demographic groups, with corrective action triggers when disparities are detected. As highlighted by a recent scoping review, telemedicine’s unintended consequences related to inequalities require systematic attention from policymakers and healthcare providers^44^.

Building on these general principles, we propose four specific, actionable measures for healthcare organisations expanding virtual care. First, integrated care systems should establish real-time equity dashboards that track RTA stratified by ethnicity, deprivation quintile, age group, and appointment modality, with pre-defined thresholds that trigger local review when disparities are detected. Second, patients from groups that did not benefit from virtual appointments in our analysis, notably those in the most deprived areas (IMD 1), Black/Black British patients, and those aged 80 years and over, should be prioritised for targeted support, including digital inclusion programmes, language interpretation services, and assisted-technology access. Third, GP referral letters should routinely capture patient communication preferences, language needs, and digital access capacity, enabling secondary care teams to allocate appointment modality more appropriately. Fourth, rather than adopting universal virtual-by-default approaches, healthcare systems should develop condition- and patient-specific eligibility criteria for virtual appointments, ensuring that efficiency gains are not achieved at the cost of equity.

This study uses a large, complete dataset of 12,474 appointments from routine NHS care, reflecting real-world practice. The four-year study period (2021-2024) captures the growth of virtual care from pandemic disruption through post-pandemic implementation. Stratified analyses across multiple demographic and clinical characteristics assessed equity systematically. Fourth, sensitivity analyses including bootstrap confidence intervals, multiple testing correction, and patient-level clustering confirmed the robustness of our findings. The study benefits from the ethnically diverse population of Northwest London, providing insights into equity implications across different ethnic groups that may be underrepresented in other settings.

The retrospective observational design prevents causal inference, and additional research (particularly prospective or experimental studies) is required to validate these findings. Whether virtual appointment allocation was clinically appropriate or biased cannot be determined from our data. No clinical outcome measures (HbA1c control, complications, hospital admissions, costs) were included in our study. This study was conducted in the northwest London GP-registered population and findings may not generalise to other populations or non-urban settings. Our study analysed the T2D population only, so findings may differ for other long-term conditions. Finally, the data of this study lacked potential mediators of disparities such as digital literacy, language proficiency, and internet access.

A further key limitation is that appointment modality was not randomly assigned. Clinicians likely allocated virtual appointments to patients judged suitable — for example, those with less complex presentations, adequate digital access, or prior in-person assessment — while in-person appointments may have been reserved for more complex or urgent cases. These selection factors may themselves be associated with waiting times (e.g., lower clinical urgency may permit more scheduling flexibility, leading to both virtual allocation and shorter RTA), creating selection bias that our observational design cannot eliminate. In addition, several variables likely to influence both modality allocation and RTA were not available in our dataset, including referral urgency, digital literacy, language proficiency, patient preference, and provider-level scheduling behaviour. The absence of these variables limits our ability to fully account for confounding, particularly when interpreting the equity findings. Accordingly, the observed differences in RTA between virtual and in-person appointments should be interpreted as associations rather than causal effects. Future research using quasi-experimental designs — such as instrumental variable analysis, difference-in-differences, or natural experiments exploiting policy changes in virtual care delivery — would be needed to establish causal effects.

Finally, this study focused exclusively on referral-to-appointment time as a measure of timeliness. While timely access is an important dimension of quality care, shorter waiting times do not necessarily translate into improved clinical outcomes. Future studies should examine whether reductions in RTA with virtual care are accompanied by improvements in HbA1c control, complication rates, patient satisfaction, or other patient-relevant outcomes.

Virtual appointments were associated with shorter referral-to-appointment times for Type 2 diabetes by up to 44%, but these benefits varied by different patient groups. However, the observational nature of this analysis means that causal relationships cannot be inferred, and additional research (particularly prospective or experimental studies) is required to validate these findings. Substantial differences between groups were also observed, highlighting the importance of healthcare organisations monitoring equity to ensure that potential benefits are distributed fairly across all populations.

## Supplementary information

Supplementary Materials

## Data Availability

The data that support the findings of this study are not publicly available. Request to access the datasets used in the paper via secure environment can be made via the WSIC Data Access Committee, subject to relevant approvals and data governance. Access requires institutional agreements and data cannot be shared publicly.
