# Supplementary material for: Impact of virtual appointments on referral-to-appointment times for type 2 diabetes patients in Northwest London
Source: Npj Health Syst. 2026 Jul 1;3:55. doi: 10.1038/s44401-026-00112-0 (PMC13354150; doi:10.1038/s44401-026-00112-0)
Supplement: Supplementary file 1 — Supplementary Materials [file 44401_2026_112_MOESM1_ESM.docx]

**Supplementary Materials**

**Supplementary Table S1: Variables extracted from WSIC database**

| **Category** | **Variable Name** | **Description/Categories** |
| --- | --- | --- |
| **Demographic** | Age | Age at referral (years); categorised as 18-39, 40-59, 60-79, 80+ years for analysis |
|  | Gender | Male/Female |
|  | Ethnicity | 18 Office for National Statistics (ONS) categories collapsed into six groups: White, Asian/Asian British, Black/Black British, Mixed, Other, Unknown |
|  | Deprivation | Index of Multiple Deprivation (IMD) quintile (1=most deprived to 5=least deprived) derived from residential postcode |
| **Clinical** | Charlson Comorbidity Index | Validated weighted scoring system incorporating 17 comorbid conditions; scored as integer from 0 upwards |
|  | Long-Term Conditions (LTCs) | Total number of LTCs recorded; categorised as 1, 2, or 3+ conditions for analysis |
|  | Specific Diagnoses | Diabetes-related complications, cardiovascular disease, chronic kidney disease, cancer (ICD-10 coded) |
| **Appointment** | Referral Date | Date referral request was received by secondary care |
|  | Appointment Date | Date of first specialist appointment |
|  | Appointment Mode | Virtual (telephone consultation from patient's location) or In-person (In-person consultation at secondary care setting) |
|  | Appointment Type | First or follow-up appointment |
|  | Medical Specialty | Specialty code for service delivering the appointment (e.g., Endocrinology, General Medicine, Diabetic Medicine, Non-consultant professional, Other) |

**Supplementary Table S2: Δ Median RTA by Patient Characteristics**

| **Characteristic** | **Median RTA** (days) | | **Δ Median RTA** = (in-person − Virtual) | |  |  | | |
| --- | --- | --- | --- | --- | --- | --- | --- | --- |
|  | **In-person** | **Virtual** | (days)* | **Difference (%)**** | | | **p-value** |  |
| **Gender** |  |  |  | |  |  | |  |
| Male | 32 | 13.5 | +18.5 | | +57.8% | <0.001 | |  |
| Female | 44 | 27 | +17 | | +38.6% | <0.001 | |  |
| **Age Group** |  |  |  | |  |  | |  |
| 18-39 years | 52 | 60 | **-8** | | **-15.4%** | 0.42 | |  |
| 40-59 years | 35 | 20 | +15 | | +42.9% | <0.001 | |  |
| 60-79 years | 34 | 12 | +22 | | +64.7% | <0.001 | |  |
| 80+ years | 42 | 36 | +6 | | +14.3% | 0.08 | |  |
| **Ethnicity** |  |  |  | |  |  | |  |
| Asian/Asian British | 43 | 17 | +26 | | +60.5% | <0.001 | |  |
| White | 27 | 14 | +13 | | +48.1% | <0.001 | |  |
| Black/Black British | 42 | 46 | **-4** | | **-9.5%** | 0.33 | |  |
| Mixed | 35 | 45.5 | **-10.5** | | **-30.0%** | 0.21 | |  |
| Other | 35 | 28 | +7 | | +20.0% | 0.08 | |  |
| **IMD Quintile** |  |  |  | |  |  | |  |
| 1 (most deprived) | 39 | 16 | +23 | | +59.0% | <0.001 | |  |
| 2 | 36 | 18 | +18 | | +50.0% | <0.001 | |  |
| 3 | 37 | 20 | +17 | | +45.9% | <0.001 | |  |
| 4 | 36 | 21 | +15 | | +41.7% | <0.001 | |  |
| 5 (least deprived) | 33 | 6 | +27 | | +81.8% | <0.001 | |  |
| **Number of LTCs** |  |  |  | |  |  | |  |
| 1 | 44 | 11 | +33 | | +75.0% | <0.001 | |  |
| 2 | 37 | 17 | +20 | | +54.1% | <0.001 | |  |
| 3+ | 35 | 23 | +12 | | +34.3% | <0.001 | |  |

*Positive values indicate shorter RTA times for virtual appointments (better timeliness).
**Positive percentages indicate reduction in RTA with Virtual appointments.

Note: All comparisons represent pooled data across 2021-2024 study period, showing overall differences between appointment modes by patient subgroup.

**Supplementary Table S3: Regression Analysis of Referral-to-Appointment Time**

**MODEL 1: Additive Model**

RTA time ~ Charlson Comorbidity Score + Appointment Mode

| Variable | β (days) | SE | p-value | 95% CI |
| --- | --- | --- | --- | --- |
| Charlson Comorbidity Score | -1.20 | 0.36 | <0.001 | -1.91, -0.49 |
| Appointment Mode (Virtual vs In-person) | -18.19 | 3.42 | <0.001 | -24.90, -11.48 |

**Model 2: Interaction Model**

RTA time ~ Charlson Comorbidity Score × Appointment Mode

| Variable | β (days) | SE | p-value | 95% CI |
| --- | --- | --- | --- | --- |
| Charlson Comorbidity Score | -1.15 | 0.38 | 0.003 | -1.89, -0.41 |
| Appointment Mode (Virtual vs In-person) | -16.58 | 5.64 | 0.003 | -27.65, -5.51 |
| Charlson Score × Remote Appointment | -0.37 | 1.02 | 0.719 | -2.37, 1.63 |
| CI = Confidence Interval; SE = Standard Error | | | |  |

Note: Both models controlled for Charlson Comorbidity Index (continuous) and appointment mode (binary: remote vs face-to-face). Negative β coefficients for Charlson score indicate that patients with higher comorbidity burden had shorter waiting times. The non-significant interaction term (p=0.719) indicates that the relationship between comorbidity and RTA time did not differ by appointment mode. Very low R² values (<0.01) indicate that RTA time is predominantly determined by factors beyond comorbidity burden and appointment mode. Sample size: n=12,474 appointments.

**Supplementary 2 STROBE Statement Checklist for cohort studies**

| Item No | Recommendation | Reported (Page/Section) | Comments |
| --- | --- | --- | --- |
| Title and abstract | | | |
| 1a | Indicate the study's design with a commonly used term in the title or the abstract | Title/Abstract | 'retrospective cohort analysis' |
| 1b | Provide in the abstract an informative and balanced summary of what was done and what was found | Abstract | Structured abstract includes background, methods, results, and conclusions |
| Introduction | | | |
| 2 | Explain the scientific background and rationale for the investigation being reported | Background | Discusses adoption of Virtual care in type 2 diabetes management and equity concerns |
| 3 | State specific objectives, including any prespecified hypotheses | Background | Objective stated: assess whether RTA improvements with Virtual appointments were equitably distributed |
| Methods | | | |
| Study design | | | |
| 4 | Present key elements of study design early in the paper | Methods | Retrospective cohort analysis clearly stated |
| Setting | | | |
| 5 | Describe the setting, locations, and relevant dates, including periods of recruitment, exposure, follow-up, and data collection | Methods: Study Design and Population | Northwest London, January 2021 - December 2024. Data extracted from WSIC database |
| Participants | | | |
| 6a | Give the eligibility criteria, and the sources and methods of selection of participants. Describe methods of follow-up | Methods: Study Design and Population | Inclusion: adults ≥18 years with T2D, first specialist appointment, complete referral dates. Exclusion: referral dates after appointment dates (data errors), RTA >365 days (outliers) |
| 6b | For matched studies, give matching criteria and number of exposed and unexposed | N/A |  |
| Variables | | | |
| 7 | Clearly define all outcomes, exposures, predictors, potential confounders, and effect modifiers. Give diagnostic criteria, if applicable | Methods: Data Sources and Variables, Supplementary Table S1 | RTA defined as primary outcome. Variables presented in Supplementary Table S1 including demographics and appointment mode. |
| Data sources/measurement | | | |
| 8 | For each variable of interest, give sources of data and details of methods of assessment (measurement). Describe comparability of assessment methods if there is more than one group | Methods: Data Sources and Variables | WSIC database described. ICD-10 coding for T2D identification. Electronic health records for referral pathway data |
| Bias | | | |
| 9 | Describe any efforts to address potential sources of bias | Discussion: Limitations | Acknowledged observational design limitations, inability to determine clinical appropriateness of Virtual allocation, and potential selection bias |
| Study size | | | |
| 10 | Explain how the study size was arrived at | Results | From 13,198 initial records, 724 (5.5%) were excluded due to data entry error (referral dates after appointment dates, n=689, 5.2%), or extreme outliers (RTA >365 days, n=35, 0.3%). Total 12,474 appointments for 3,088 patients. Complete cohort of eligible patients during study period |
| Quantitative variables | | | |
| 11 | Explain how quantitative variables were handled in the analyses. If applicable, describe which groupings were chosen and why | Methods: Statistical Analysis | RTA reported as median with IQR due to right-skewed distribution. Age groups: 18-39, 40-64, 65+ years. IMD quintiles for deprivation |
| Statistical methods | | | |
| 12a | Describe all statistical methods, including those used to control for confounding | Methods: Statistical Analysis | Wilcoxon rank-sum tests with Benjamini-Hochberg false discovery rate correction compared RTA time between virtual and in-person appointments. Stratified analyses by demographics. p<0.05 significance threshold |
| 12b | Describe any methods used to examine subgroups and interactions | Methods: Statistical Analysis | Stratified analyses by age, gender, ethnicity, deprivation, and clinical characteristics. Year-by-year temporal analysis |
| 12c | Explain how missing data were addressed | Methods: Statistical Analysis | 5% of records with missing age, gender, ethnicity, or LTCs excluded from stratified analyses. Complete data required for referral/appointment dates |
| 12d | If applicable, explain how loss to follow-up was addressed | N/A |  |
| 12e | Describe any sensitivity analyses | Methods: Sensitivity Analyses | Two sensitivity analyses: (1) Bootstrap CIs with patient-level resampling, (2) Patient-level adjusted analysis (n=3,289) |
| Data access and cleaning methods | | | |
| * | Provide information on data access and cleaning methods | Methods: Data Availability | WSIC database access via Data Access Committee. Not publicly available, requires institutional agreements. Data cleaning: excluded 724 records (5.5%) - 689 data entry errors, 35 outliers |
| Results | | | |
| Participants | | | |
| 13a | Report numbers of individuals at each stage of study, e.g.: numbers potentially eligible, examined for eligibility, confirmed eligible, included in the study, completing follow-up, and analysed | Results: Study Population | 13,198 initial records; 12,474 appointments (3,088 patients) included after exclusions. 90.3% in-person, 9.7% Virtual |
| 13b | Give reasons for non-participation at each stage | Methods: Study Design and Population | 724 records (5.5%) excluded: 689 (5.2%) with referral dates after appointment dates (data errors), 35 (0.3%) outliers with RTA >365 days |
| 13c | Consider use of a flow diagram | Not included |  |
| Descriptive data | | | |
| 14a | Give characteristics of study participants (e.g. demographic, clinical, social) and information on exposures and potential confounders | Results: Study Population, Table 2 | Demographics presented: age, gender, ethnicity, deprivation, comorbidities. Comparison between Virtual and in-person groups |
| 14b | Indicate number of participants with missing data for each variable of interest | Methods: Statistical Analysis | 5% of records had missing data on age, gender, ethnicity, or LTCs and were excluded from stratified analyses |
| 14c | Summarise follow-up time (e.g., average and total amount) | N/A |  |
| Outcome data | | | |
| 15 | Report numbers of outcome events or summary measures over time | Results: RTA Comparisons | Median RTA reported overall and stratified by demographics and year. Temporal trends 2021-2024 presented |
| Main results | | | |
| 16a | Give unadjusted estimates and, if applicable, confounder-adjusted estimates and their precision (e.g., 95% confidence interval). Make clear which confounders were adjusted for and why they were included | Results: RTA Comparisons; Methods: Sensitivity Analyses | Median RTA differences reported with p-values. Bootstrap 95% CIs with patient-level clustering provided in sensitivity analyses |
| 16b | Report category boundaries when continuous variables were categorised | Methods/Results | Age groups: 18-39, 40-64, 65+ years. IMD quintiles: 1 (least deprived) to 5 (most deprived) |
| 16c | If relevant, consider translating estimates of relative risk into absolute risk for a meaningful time period | Results | Absolute differences in days reported alongside percentage reductions |
| Other analyses | | | |
| 17 | Report other analyses done e.g. analyses of subgroups and interactions, and sensitivity analyses | Results: Equity Analysis; Methods/Results: Sensitivity Analyses | Extensive stratified analyses by demographics. Temporal analysis by year. Three sensitivity analyses conducted and reported |
| Discussion | | | |
| 18 | Summarise key results with reference to study objectives | Discussion | Key findings summarised: overall RTA improvements with Virtual care, but inequitable distribution across demographic groups |
| 19 | Discuss limitations of the study, taking into account sources of potential bias or imprecision. Discuss both direction and magnitude of any potential bias | Discussion: Limitations | Comprehensive limitations discussed: observational design, single healthcare system, lack of clinical outcomes, potential allocation bias, missing mediators |
| 20 | Give a cautious overall interpretation of results considering objectives, limitations, multiplicity of analyses, results from similar studies, and other relevant evidence | Discussion | Balanced interpretation provided with policy implications and call for equity-focused implementation. Sensitivity analyses address multiplicity |
| 21 | Discuss the generalisability (external validity) of the study results | Discussion: Limitations | Acknowledged: single healthcare system (NW London), urban/ethnically diverse population may not represent rural settings, T2D-specific findings |
| Other information | | | |
| 22 | Give the source of funding and the role of the funders for the present study and, if applicable, for the original study on which the present article is based | Funding | NIHR ARC NWL independent research. RA: Saudi Ministry of Education. ALN: NIHR NWL PSRC & Imperial NIHR BRC. Views are authors', not NIHR/DHSC |
| * | Provide information on ethical approval | Ethics and Data Governance | NW London Sub-Data Research Access Group approval: 14 June 2024, using anonymised routine data per UK HRA guidelines (no separate REC approval required) |
